# Supplementary material for: Large-scale network analysis captures biological features of bacterial plasmids
Source: Nat Commun. 2020 May 15;11:2452. doi: 10.1038/s41467-020-16282-w (PMC7229196; doi:10.1038/s41467-020-16282-w)
Supplement: Supplementary file 3 — Description of Additional Supplementary Files [file 41467_2020_16282_MOESM3_ESM.docx]

**Description of Additional Supplementary Files**

**File name:** Supplementary Data 1

**Description:** A large table containing metadata for all complete bacterial plasmid sequences analysed in this study, including accession number, plasmid name, GC content, sequence length, replicon and MOB type, and host organism. In addition, the information about the plasmid clique allocations is included, as well as the accession numbers and positions for retrieving sequences of the corresponding candidate replicon genes.
