# Supplementary material for: Large-scale network analysis captures biological features of bacterial plasmids
Source: Nat Commun. 2020 May 15;11:2452. doi: 10.1038/s41467-020-16282-w (PMC7229196; doi:10.1038/s41467-020-16282-w)
Supplement: Supplementary file 5 — Reporting Summary [file 41467_2020_16282_MOESM5_ESM.pdf]

nature research

Corresponding author(s): Mislav Acman and Francois Balloux

Last updated by author(s): Apr 13, 2020

Reporting Summary

Nature Research wishes to improve the reproducibility of the work that we publish. This form provides structure for consistency and transparency in reporting. For further information on Nature Research policies, see Authors & Referees and the Editorial Policy Checklist .

Statistics

For all statistical analyses, confirm that the following items are present in the figure legend, table legend, main text, or Methods section.

n/a	Confirmed	
[ ]	[x]	The exact sample size (*n*) for each experimental group/condition, given as a discrete number and unit of measurement
[x]	[ ]	A statement on whether measurements were taken from distinct samples or whether the same sample was measured repeatedly
[ ]	[x]	The statistical test(s) used AND whether they are one- or two-sided<br>*Only common tests should be described solely by name; describe more complex techniques in the Methods section.*
[ ]	[x]	A description of all covariates tested
[ ]	[x]	A description of any assumptions or corrections, such as tests of normality and adjustment for multiple comparisons
[ ]	[x]	A full description of the statistical parameters including central tendency (e.g. means) or other basic estimates (e.g. regression coefficient) AND variation (e.g. standard deviation) or associated estimates of uncertainty (e.g. confidence intervals)
[ ]	[x]	For null hypothesis testing, the test statistic (e.g. *F*, *t*, *r*) with confidence intervals, effect sizes, degrees of freedom and *P* value noted<br>*Give P values as exact values whenever suitable.*
[x]	[ ]	For Bayesian analysis, information on the choice of priors and Markov chain Monte Carlo settings
[ ]	[x]	For hierarchical and complex designs, identification of the appropriate level for tests and full reporting of outcomes
[x]	[ ]	Estimates of effect sizes (e.g. Cohen's *d*, Pearson's *r*), indicating how they were calculated

Our web collection on statistics for biologists contains articles on many of the points above.

Software and code

Policy information about availability of computer code

Data collection	Complete plasmid sequences and metadata were downloaded from publicly available NCBI's RefSeq release repository (accessed on 26/09/2018). No software was used for data download. Information about the taxonomic hierarchy of plasmid bacterial hosts was obtained using ETE 3 Python toolkit
Data analysis	The following software have been used in data analysis: MOBtyping (https://github.com/AlexOrlek/MOBtyping), NCBI's BLAST+ software (v2.7.1), Prokka (v1.13.3), Roary (v3.12.0), Uniprot's 'Retrieve/ID Mapping' online tool (https://www.uniprot.org/uploadlists/), Bindash (v0.2.1), Cytoscape (v3.7.1), OSLOM (v2.5) and NMI R package (v2.0). The scripts used to construct a dataset of complete bacterial plasmids, annotate the plasmid sequences, score pairwise JI similarity and perform clique detection with OSLOM are available on: https://github.com/macman123/plasmid_network_analysis

For manuscripts utilizing custom algorithms or software that are central to the research but not yet described in published literature, software must be made available to editors/reviewers. We strongly encourage code deposition in a community repository (e.g. GitHub). See the Nature Research guidelines for submitting code & software for further information.

Data

Policy information about availability of data

All manuscripts must include a data availability statement. This statement should provide the following information, where applicable:

- Accession codes, unique identifiers, or web links for publicly available datasets
- A list of figures that have associated raw data
- A description of any restrictions on data availability

All data used to perform this analysis is available in a public repository: ftp://ftp.ncbi.nlm.nih.gov/refseq/release/plasmid/. The accession codes of all plasmid sequences used in the analysis are available in Supplementary Table 1 along with the accompanying metadata. Additional databases used in the data analysis are: PlasmidFinder replicon database (v2018-09-04; https://bitbucket.org/genomicepidemiology/plasmidfinder_db/src/master/), hamap2go mapping table (v2019/05/04; http://current.geneontology.org/ontology/external2go/hamap2go)

Field-specific reporting

Please select the one below that is the best fit for your research. If you are not sure, read the appropriate sections before making your selection.

- [x] Life sciences
- [ ] Behavioural & social sciences
- [ ] Ecological, evolutionary & environmental sciences

For a reference copy of the document with all sections, see nature.com/documents/nr-reporting-summary-flat.pdf

Life sciences study design

All studies must disclose on these points even when the disclosure is negative.

Sample size	Overall, the dataset contained 10,696 complete bacterial plasmid sequences.
Data exclusions	Plasmid sequences used in the analysis were chosen based on the following criteria:<br>1) They were obtained from a bacterial host<br>2) They had a title annotation matching the regular expression: "plasmid.*complete sequence"
Replication	Accession codes and the metadata used in the analysis can be found in Supplementary Data 1. Source data underlying the figures are provided. The scripts used to perform similarity scoring and clique detection were made available on GitHub. Methods used to assess biological significance of plasmid cliques are described in Methods and Results section.
Randomization	The randomization is not applicable due to design of this study.
Blinding	The blinding is not applicable due to design of this study.

Reporting for specific materials, systems and methods

We require information from authors about some types of materials, experimental systems and methods used in many studies. Here, indicate whether each material, system or method listed is relevant to your study. If you are not sure if a list item applies to your research, read the appropriate section before selecting a response.

Materials & experimental systems

n/a	Involved in the study	
[x]	[ ]	Antibodies
[x]	[ ]	Eukaryotic cell lines
[x]	[ ]	Palaeontology
[x]	[ ]	Animals and other organisms
[x]	[ ]	Human research participants
[x]	[ ]	Clinical data

Methods

n/a	Involved in the study	
[x]	[ ]	ChIP-seq
[x]	[ ]	Flow cytometry
[x]	[ ]	MRI-based neuroimaging
